# Advances in Laser-Induced Graphene for Flexible Sensors

**DOI:** 10.3390/ma19091851

**Published:** 2026-04-30

**Authors:** Lishuang Lin, Huiqi Yang, Haifeng Gao, Jiaqi Wang, Longhui Zheng, Zimin Hong, Lixin Wu

**Affiliations:** 1 College of Chemistry, Fuzhou University, Fuzhou 350108, China; 2 Fujian Key Laboratory of Nanomaterials, Fujian Institute of Research on the Structure of Matter, Chinese Academy of Sciences, Fuzhou 350002, China; 3 Fujian College, University of Chinese Academy of Sciences, Fuzhou 350002, China; 4 Putian Institute of New Functional Materials Technology, Putian 351100, China

**Keywords:** laser-induced graphene, sensing mechanism, performance optimization, strain sensor, temperature sensor

## Abstract

Laser-induced graphene (LIG) is a porous carbon material produced in situ by direct laser irradiation of carbon-containing precursors. With its three-dimensional porous structure, high electrical conductivity, facile patternability, low cost, and environmentally friendly fabrication, LIG has attracted growing interest for flexible sensing applications. It shows strong potential in wearable electronics, health monitoring, human–machine interaction, environmental sensing, and intelligent robotics. Although LIG-based sensors have demonstrated excellent performance in mechanical and thermal signal detection, a systematic review of their basic materials, formation mechanisms, sensing principles, structural design, performance optimization, and applications remains limited. This review first summarizes the fundamental materials, processing parameters, and formation principles of LIG, and then highlights recent progress in LIG-based strain and temperature sensors, focusing on sensing mechanisms, key performance indicators, optimization strategies, and research status. The main challenges for practical application are also discussed. These include limited material uniformity and fabrication reproducibility, signal coupling and interference in multifunctional devices, and issues of process compatibility and packaging reliability. Future directions for high-performance, integrated, and scalable LIG sensors are then.

## 1. Introduction

With the rapid development of information technology, materials science, and intelligent manufacturing, electronic devices are continuously evolving from conventional rigid formats toward flexible, lightweight, and wearable forms. As a result, flexible electronics has become an important development direction in the next generation of electronic information technologies. Compared with conventional rigid electronic devices based primarily on silicon and metallic materials, flexible electronic devices, including flexible sensors [1,2,3], supercapacitors [4,5], flexible heaters [6], and nanogenerators [7], exhibit excellent mechanical flexibility, lightweight characteristics, and good electrical stability. They can maintain stable electrical performance under bending, stretching and even complex deformation conditions. This makes them promising for application in health monitoring, wearable electronics, human–machine interaction, and flexible integrated systems.

Among the various flexible electronic devices, wearable sensors have attracted extensive attention in recent years as key components for human-information perception, acquisition, and interaction. These devices can continuously collect physiological signals such as heart rate, respiration rate, body temperature, and electromyographic signals [8]. At the same time, they enable real-time monitoring of environmental information, including pressure, temperature, and humidity [9,10,11]. These signals can then be converted into electrical outputs for subsequent analysis and processing. In particular, strain sensors can effectively detect mechanical signals such as human motion, joint bending, and subtle deformation, while temperature sensors allow real-time monitoring of body-surface and environmental thermal signals. As such, these two types of sensors are core components for constructing high-performance wearable health-monitoring systems. Therefore, the development of flexible strain and temperature sensors with high sensitivity, high stability, good flexibility, and excellent wearing comfort is of great research significance.

As an essential component of wearable sensors, flexible substrates play a crucial role in determining the overall device performance, signal stability, and user wearing experience. Traditional flexible substrate materials mainly include polymers such as polyimide (PI) [12] and polydimethylsiloxane (PDMS) [13]. Although these materials possess good flexibility and processability, they still suffer from certain limitations in terms of comfort and long-term conformal contact with human skin. In contrast, textile substrates offer inherent softness, light weight, and superior wearing comfort, making them more suitable for the construction of skin-conformal wearable sensors. Therefore, imparting sensing functionality to textile materials has become an important research direction in the fields of flexible electronics and intelligent textiles.

In addition, the rational selection and design of sensing materials play a decisive role in improving device performance. Owing to its unique two-dimensional structure, high carrier mobility, outstanding mechanical strength, excellent flexibility, and large specific surface area [14], graphene is widely regarded as an ideal material for constructing high-performance flexible sensors. In particular, LIG technology enables the in situ fabrication of porous graphene on flexible substrates, combining facile processing with excellent performance. It thus provides a new technical route for the integrated fabrication of textile-based flexible sensors. However, current LIG-based sensors still suffer from insufficient sensitivity, which seriously limits their application in weak-signal detection and practical wearable systems.

On this basis, this review systematically summarizes the research progress of LIG for flexible wearable sensors. First, the material basis and precursor types of LIG are introduced. Then, the preparation methods and formation mechanisms of LIG are summarized. Subsequently, the application status, performance regulation strategies, and representative advances of LIG in flexible strain and temperature sensors are discussed separately. Finally, the major challenges in this field and future development trends are summarized and prospected.

## 2. Overview of Laser-Induced Graphene

**Figure 1:**
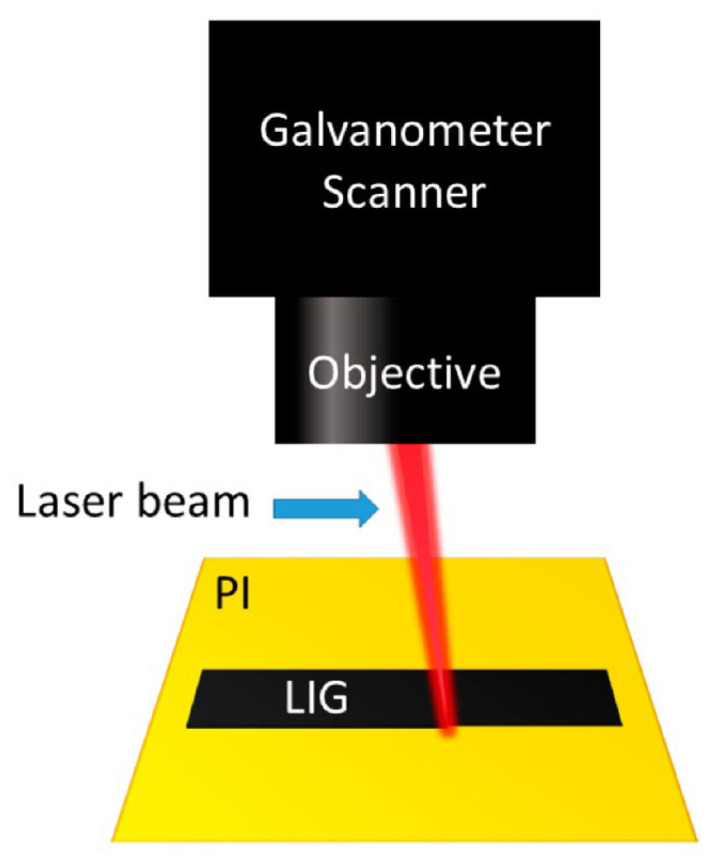
Schematic illustration of LIG [24].

In 2014, Lin et al. [15] first introduced laser-induced graphene (LIG). As shown in Figure 1, LIG refers to a class of porous conductive carbon materials that are formed in situ from carbon-containing precursors through instantaneous localized pyrolysis, carbonization, and graphitization under laser irradiation [16]. With further research, Ye et al. [17] have provided a more systematic understanding of the fabrication mechanisms, structural characteristics, and application potential of LIG. Compared with conventional graphene fabrication methods, such as mechanical exfoliation [18], chemical vapor deposition [19], and oxidation–reduction methods [20], LIG offers several distinctive advantages, including a simplified fabrication process, transfer-free processing, direct patterning capability, and compatibility with various flexible substrates. Consequently, it has attracted extensive attention in fields such as flexible electronics [21], energy storage devices [22], and sensors [23]. In particular, for flexible sensors, LIG combines good electrical conductivity, mechanical flexibility, and tunable structural features, providing an important material foundation for high-performance sensing layers.

The formation of LIG is essentially governed by the combined effects of precursor properties and laser processing conditions. Specifically, the chemical composition, thermal stability, carbon content, and decomposition pathway of the precursor determine the carbonization behavior, graphitization degree, pore-structure evolution, and surface chemical state under laser irradiation. Processing parameters such as laser power, scanning speed, focal distance, and ambient atmosphere further influence localized heat accumulation and material conversion. These factors ultimately determine the structural characteristics and performance of LIG. Therefore, systematically clarifying precursor types and their formation-regulation rules is of great significance for understanding the formation mechanism of LIG and achieving controllable structural fabrication.

### 2.1. Precursor Materials

According to their sources and compositional characteristics, the precursors currently used for LIG fabrication can generally be classified into three categories: polymer precursors, natural-material precursors, and composite precursors.

Polymer precursors are the most widely used class of materials for LIG fabrication, among which polyimide is the most representative [25]. In addition, PI possesses excellent thermal stability, mechanical properties, and flexibility, making it widely used in flexible electronics and sensing applications. Beyond PI, polyether ether ketone (PEEK) [26], polyacrylonitrile (PAN) [27], and some other polymers containing aromatic structures can also serve as precursors for LIG fabrication.

With the development of green manufacturing and sustainable materials research, natural-material precursors have gradually become an important direction in LIG fabrication. Wood [28], fibrous textiles [29], paper [30], cellulose [31], lignin [32], and other biomass-derived materials are considered promising LIG precursors because of their abundant availability, low cost, and high carbon content. Compared with conventional polymeric materials, natural materials usually possess intrinsic porous structures, fibrous networks, and hierarchical features. These structural characteristics facilitate the formation of multiscale pores and complex surface morphologies during laser induction, thereby providing favorable conditions for enhancing sensing performance.

To further improve the structural controllability and functional diversity of LIG, composite precursors have attracted extensive attention in recent years. Composite precursors are typically constructed by introducing functional components, such as conductive fillers, metal or metal-oxide particles, inorganic salts, and carbon-based nanomaterials, into a base precursor. Through the synergistic effects of multiple components, the laser-induced process can be regulated to tailor the electrical conductivity, graphitization degree, pore structure, and interfacial chemical state of LIG. Compared with single-component precursors, composite precursors offer greater design flexibility. On the one hand, sacrificial templates or phase-separated components help generate more diverse hierarchical pore structures [33], thereby enhancing the sensitivity of materials to strain, pressure, or temperature stimuli. On the other hand, the incorporation of functional particles such as metal oxides [34] and carbon nanomaterials [35] can endow LIG with stronger interfacial regulation capability and synergistic response behavior, improving the sensitivity, linearity, or selectivity of devices. Liu et al. [36] introduced platinum acetylacetonate as a functional component into a polybenzimidazole (PBI) substrate and used laser ablation to generate a Pt/LIG composite in situ, fabricating a highly sensitive strain sensor. The device exhibited gauge factor (GF) of 45.6 (0–6%), 269.5 (6–16%), and 489.3 (16–20%) across different strain ranges, demonstrating the potential of composite precursors to enhance both the sensitivity and structural controllability of LIG-based sensors.

### 2.2. Flexible Substrates

Flexible substrates not only support LIG formation and device construction but also influence the microstructure, interfacial adhesion, and sensing performance of LIG-based devices. Compared with conventional rigid substrates, flexible substrates generally exhibit bendability, stretchability, light weight, and ease of integration, making them more suitable for application scenarios such as wearable electronics, electronic skin, and smart textiles. For LIG-based flexible sensors, the thermal stability, surface morphology, and mechanical properties of the substrate, together with its interfacial interactions with the precursor or conductive layer, directly affect the carbonization behavior during laser induction, the formation of conductive networks, and the device stability under repeated deformation.

Common flexible substrates for LIG-based sensors mainly include polyimide (PI), polyethylene terephthalate (PET) [37], polydimethylsiloxane (PDMS) [38], thermoplastic polyurethane (TPU) [39], and paper-based materials [40]. Among them, PI has been widely used as a typical substrate for direct laser induction of LIG because of its excellent heat resistance, chemical stability, and mechanical performance. Polyester films such as PET exhibit good transparency and flexibility, although their thermal resistance is relatively limited. Elastomers such as PDMS and TPU show distinct advantages in wearable devices due to their excellent stretchability and skin conformability. Paper-based materials have also been used in the construction of flexible electronic devices because of their wide availability, low cost, and ease of processing, although their moisture resistance and long-term stability remain limited. Overall, different flexible substrates differ in thermal stability, surface morphology, and device integration modes, and these differences determine their applicable scenarios and performance characteristics in LIG sensors.

Compared with film-based and elastomeric substrates, textile substrates possess more complex fibrous network structures and more prominent wearable characteristics, making them a particularly distinctive class of substrate materials for LIG-based flexible sensors. Textiles are typically formed by interweaving fibers or yarns and therefore exhibit inherent porosity, flexibility, and breathability [41]. These features provide a comfortable wearing experience while offering abundant interfacial contact areas for conductive-network construction. Owing to these characteristics, textile substrates offer advantages such as light weight, breathability, good skin conformity, and ease of integration with garments, making them particularly suitable for applications in human-motion monitoring, physiological signal acquisition, and smart textiles. However, in practical use, textiles are often subjected to repeated friction, washing, and large deformation, which imposes higher requirements on interfacial adhesion and long-term device stability.

### 2.3. Effect of Processing Parameters on the Structure of LIG

#### 2.3.1. Laser Power

**Figure 2:**
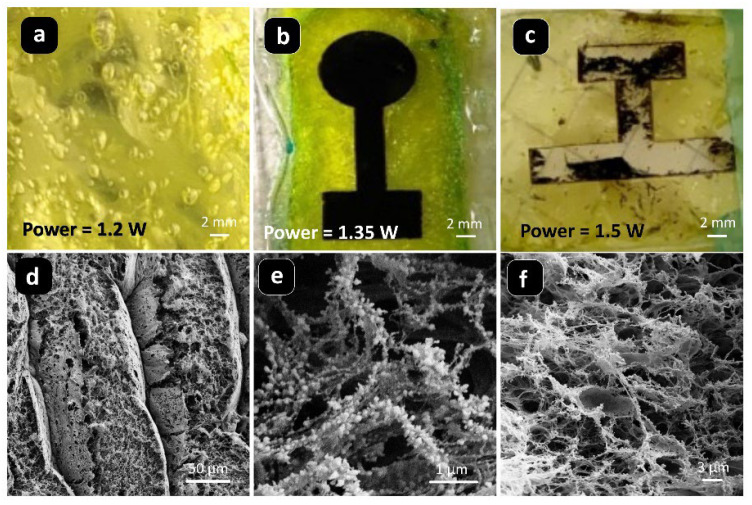
Morphology of laser-induced graphene (LIG) on chitosan films at different laser powers: (**a**) 1.2 W, (**b**) 1.35 W, (**c**) 1.5 W; SEM images of LIG on chitosan films at different laser powers: (**d**) 1.2 W, (**e**) 1.35 W, (**f**) 1.5 W [43].

Laser power determines the intensity of instantaneous heat input at the precursor surface and is therefore one of the key parameters affecting the quality of LIG formation. As shown in Figure 2, when the laser power is low (1.2 W), the degree of carbonization on the sample surface is insufficient, and the laser-induced region is discontinuous, making it difficult to form a complete and well-defined conductive pattern (Figure 2a). The cross-sectional morphology is relatively dense, and no well-developed porous structure has formed (Figure 2d). This indicates that the local temperature is still insufficient to drive adequate precursor decomposition and graphitization. When the power is increased to 1.35 W, a continuous and well-defined conductive pattern with clear boundaries can be formed on the sample surface (Figure 2b). At the microscopic level, a more developed three-dimensional porous structure is observed (Figure 2e). This suggests that an appropriate heat input facilitates precursor decomposition, the removal of non-carbon elements, and the construction of a sp^2^ carbon network, thereby promoting the formation of both the conductive network and porous structure [15]. When the power is further increased to 1.5 W, obvious ablation and structural damage appear on the sample surface, and the integrity of the pattern deteriorates significantly (Figure 2c). Although the microstructure still retains certain porous features, local collapse and disorder are observed (Figure 2f). This indicates that excessive laser power produces overly strong heat input, causing over-ablation of the material, damage to the pore walls, and even local destruction of the substrate, ultimately impairing the continuity of the conductive network and the stability of the device [42]. These results demonstrate that laser power plays a significant regulatory role in LIG formation, and an appropriate power window must be identified in practical processing to balance sufficient carbonization and structural integrity.

#### 2.3.2. Scanning Speed

Scanning speed mainly affects the laser dwell time and energy deposition per unit area. At a lower scanning speed, the precursor receives more sufficient thermal input, which is conducive to carbonization and graphitization and promotes the formation of porous structures and conductive networks. In contrast, an excessively high scanning speed tends to result in insufficient carbonization, discontinuous conductive pathways, and deteriorated electrical performance. However, an overly low scanning speed may also cause excessive local heat accumulation, inducing material ablation and pore structure instability. Therefore, scanning speed is not an isolated parameter but rather an important factor that, together with laser power, determines the effective heat input per unit area and the pathway of structural evolution.

#### 2.3.3. Processing Environment and Atmosphere

The processing environment and atmosphere also play important roles in the formation behavior of LIG. When processing is carried out in air, the carbonization and graphitization of the precursor are usually accompanied by a certain degree of oxidation. As a result, the resulting LIG surface often retains more oxygen-containing functional groups and active sites [44]. This is beneficial for enhancing surface activity and the potential for subsequent functionalization, but it may also affect the intrinsic electrical conductivity to some extent. By contrast, processing under an inert atmosphere suppresses oxidative side reactions and is more favorable for improving the graphitization degree and electrical conductivity [45], although the chemical activity of the material surface may be correspondingly weakened. In addition, the processing environment can also influence heat transfer, the escape of volatile species, and the evolution of microstructure. For composite precursors containing metal salts, metal oxides, or other active components, the processing atmosphere may even affect valence-state changes and interfacial chemical reactions of these components [46]. Therefore, atmospheric conditions not only influence the graphitization level of LIG but also directly affect its surface functional-group composition and the interfacial reaction behavior in composite systems.

#### 2.3.4. Other Parameters

In addition to laser power, scanning speed, and processing environment or atmosphere, laser wavelength, focal distance, and the number of scanning passes also significantly affect the formation of LIG. Laser wavelength determines the absorption efficiency of optical energy by the precursor. Because different materials respond differently to lasers of different wavelengths, the wavelength can influence the local heating rate, carbonization depth, and graphitization degree [47]. Focal distance is related to the laser spot size and energy distribution. Appropriate focusing conditions help achieve more uniform energy input and clearer pattern boundaries, whereas a defocused state may lead to energy dispersion, reduced resolution, or insufficient local heat input [48]. The number of scanning passes mainly affects the extent to which the precursor undergoes repeated thermal treatment. A moderate increase in the number of passes can further promote carbonization and graphitization, whereas too many passes may cause ablation, structural collapse, or substrate damage [49]. Compared with laser power and scanning speed, these parameters are more closely associated with the fine regulation of LIG microstructure, pattern quality, and local structural uniformity.

### 2.4. Formation Mechanism of Laser-Induced Graphene

#### 2.4.1. Local Reaction Mechanism

The localized thermal effect of the laser is the primary driving force for LIG formation. When a high-energy laser beam is focused on the PI surface, the material rapidly absorbs the laser energy and converts it into heat. This intensifies molecular vibrations in the polymer, accompanied by heat conduction and thermal radiation. Owing to the highly localized and transient nature of laser irradiation, the irradiated region undergoes a series of complex changes within an extremely short time, including thermal deformation, thermal expansion, melting, decomposition, and vaporization. According to differences in temperature distribution and material-state evolution, the laser-affected region can generally be divided into a thermoforming zone, a thermomelting zone, and a heat-affected zone.

Among these, the thermoforming zone is located at the center of the laser spot and exhibits the highest temperature, usually far above the melting temperature of PI and even close to the critical conditions required for graphitization. In this region, large numbers of chemical bonds, such as C=O, C-O, C-N, and C-H, are cleaved, accompanied by the release of gaseous by-products such as CH_4_, NO_2_, and N_2_ [50]. The high-temperature environment not only promotes the transformation of the polymer from disordered carbon into a sp^2^-hybridized carbon structure but also facilitates carbon-skeleton reconstruction and the formation of a graphitized network. Thermal expansion of the surface layer and the rapid escape of volatile components simultaneously drive the carbon layer to grow upward, ultimately forming a porous graphene structure with a certain thickness. In essence, this process involves the rapid removal of heteroatoms, rearrangement of the carbon skeleton, and in situ generation of a porous conductive layer under localized high-temperature conditions. The recoil pressure generated by vaporization may also cause some molten substances or fine carbon flakes to splash outward and redeposit on the surface after cooling, forming micron- or submicron-sized carbon particles near the irradiated area.

The thermomelting zone is located outside the thermoforming zone, where the temperature is generally higher than the melting point of PI but lower than the temperature required for sufficient carbonization. In this region, the material is in a locally molten or semi-molten state, and the mobility of the components is enhanced, which is favorable for homogenizing the surface structure [51]. At the same time, some oxygen-containing species decompose and release gases such as CO and CO_2_. After escaping, these gases can leave pores inside the material or induce the formation of local microcracks. Under the combined effects of thermal stress, plastic flow, and extrusion, annular wrinkles or transition-layer structures may also appear at the edges of the thermoforming zone. Therefore, although the thermomelting zone is not the primary graphitization region, it plays an important role in improving pore structure, regulating surface morphology, and forming transition layers.

The heat-affected zone lies outside the thermomelting zone. Here, the temperature is insufficient to induce significant carbonization or melting but is still high enough to cause a certain degree of thermal disturbance within the material. In this region, the polymer may undergo local stress relaxation and slight plastic deformation, whereas its macroscopic chemical composition and overall structure usually remain largely unchanged. Although this region does not directly participate in the formation of the main LIG structure, its thermal disturbance may still affect the interfacial bonding state of adjacent regions and the overall structural integrity.

#### 2.4.2. Thermal Decomposition Process

In addition to the localized thermal effect, the decomposition pathway of the precursor and the reconstruction process of the carbon skeleton also determine the structure and properties of LIG. The conversion of the precursor into LIG under laser induction is essentially a dynamic pyrolysis process, accompanied by chemical-bond cleavage, the release of volatile small molecules, and the rearrangement of the residual carbon skeleton.

**Figure 3:**
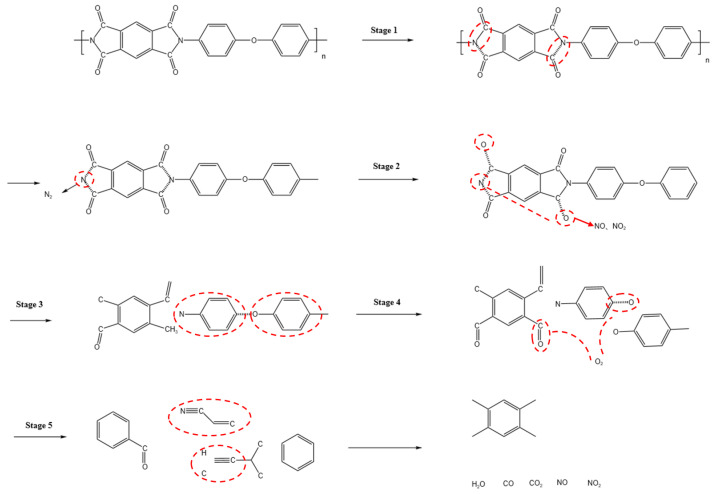
Schematic illustration of the thermal decomposition process.

As shown in Figure 3, taking PI as an example, its thermal decomposition process can be summarized as follows. First, the C-N bonds in the imide structure are cleaved, forming unstable intermediate structures. Subsequently, the carbonyl bonds are further broken and react with unstable N-containing bonds to generate nitrogen-containing gases such as NO and NO_2_. As the temperature continues to rise, some ether bonds (C-O) are cleaved, leading to the formation of benzene-ring derivatives. Under further thermal treatment, some intermediate structures undergo oxidation or rearrangement, producing oxygen-containing functional groups such as C=O and C-O. Finally, the aromatic structures decompose and carbonyl groups are removed, generating functional groups such as -C≡N and -C≡C-, along with the release of gases such as H_2_O, CO, and NO [52]. During their escape, these gases leave a number of pores within the material, endowing LIG with its characteristic porous structure.

### 2.5. Application Prospects of LIG-Based Flexible Sensors

LIG, owing to its three-dimensional continuous porous network and excellent electrical properties, offers remarkable advantages in flexible strain sensors. As shown in Table 1, LIG/Ecoflex achieves a GF of 191.55 within the 0–50% strain range, with stable cycling performance. In contrast, PDMS/carbon nanotube (CNT) and PDMS/silver nanowires (AgNWs) cover strain ranges of 30–70% but deliver low GF (<20), limiting their ability to detect subtle deformations. TPU/polydopamine (PDA)/MXene and PEDOT:PSS systems can accommodate strains of up to 150–300%, yet their GF remain moderate (17–60). Overall, LIG achieves a superior trade-off between sensitivity and strain ranges.

Beyond strain sensing, LIG shows broad potential in temperature, gas [53], pressure [54], tactile, and chemical/biological detection, owing to its high specific surface area, excellent conductivity, thermal responsiveness, and porous structure. Through surface functionalization or electrochemical modification, LIG can further identify analytes such as pH [55] and glucose [56], enabling multifunctional flexible sensing platforms. In practical applications, strain sensors capture mechanical signals associated with body motion, joint bending, and subtle deformations. Meanwhile, temperature sensors provide real-time monitoring of skin and environmental thermal variations. Together, these two modalities form core components of high-performance wearable health monitoring systems, underscoring the need for flexible sensors that combine high sensitivity, stability, mechanical compliance, and wearing comfort.

**Table 1:** Strain Performance Comparison of Various Flexible Sensing Materials.

Sensing Material	GF	Detection Range	Response/Recovery Time/ms	Number of Cycles	Reference
LIG/Ecoflex	191.55	0–50%	70/-	1500	[57]
PDMS/CNT	4.36	0–30%	-	100	[58]
PDMS/AgNWs	14	0–70%	-	-	[59]
TPU/PDA/MXene	57.15	0–300%	0.3/-	1000	[60]
PEDOT:PSS	17.1	0–150%	-	10,000	[61]

Common non-resistive LIG sensing modes include capacitive and piezoelectric types. Among them, capacitive sensors typically rely on multilayer structures, which complicate fabrication and increase vulnerability to environmental noise. While piezoelectric sensors respond only to transient loads and thus cannot support continuous monitoring. Resistive sensors, by comparison, are simple to fabricate and easy to read out, while also achieving high sensitivity across a wide detection range. Temperature sensors are mainly classified into resistive and thermoelectric types. Thermoelectric sensors depend on temperature gradients; the resulting signals are weak and susceptible to external disturbance. Resistive counterparts instead offer superior signal stability and suitability for wearable integration.

Therefore, this paper focuses on resistive strain and temperature sensors, which are systematically discussed in the following sections.

## 3. Research Progress in LIG-Based Resistive Strain Sensors

### 3.1. Sensing Mechanisms of Strain Sensors

#### 3.1.1. Geometric Effect and Intrinsic Piezoresistive Effect

Resistive strain sensors are typically composed of a flexible substrate and a conductive sensing material. The flexible substrate endows the device with good bendability and stretchability, while the conductive sensing material is responsible for converting external mechanical deformation into detectable resistance signals [62,63].

**Figure 4:**
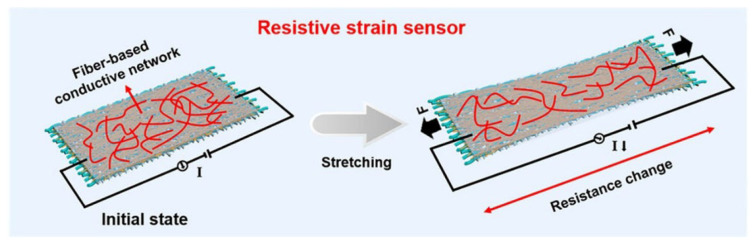
Schematic illustration of the working mechanism of the resistive strain sensor [66].

From a mechanistic perspective, the resistance response mainly arises from two aspects: the geometric effect and the intrinsic piezoresistive effect. The geometric effect can be expressed as follows [64]:

(1) $$R=\rho \frac{L}{A}$$

where *R* is the resistance, *ρ* is the resistivity of the material, *L* is the length of the conductor, and *A* is the cross-sectional area of the conductor. As shown in Figure 4, effective contact is maintained among the conductive components, forming a continuous conductive network that gives the device a stable initial resistance. When the sensor is subjected to tensile strain, the sensing layer elongates along the loading direction, the conductive pathways are extended, and the cross-sectional area decreases, leading to an increase in device resistance.

In addition to the geometric effect, resistive strain sensors may also be influenced by the intrinsic piezoresistive effect. The intrinsic piezoresistive effect refers to the phenomenon in which external mechanical stress causes a change in the resistivity of the material itself. Its relative resistance change can be expressed as follows [65]:

(2) $$\frac{\Delta R}{R_{0}}=\left(1+2v\right)\varepsilon +\frac{\Delta \rho }{\rho_{0}}$$

where Δ*R* and *R*_0_ represent the resistance change and the initial resistance, respectively, ν is Poisson’s ratio, ε is the applied strain, and Δ*ρ*/*ρ*_0_ denotes the relative change in the resistivity of the material. Among these terms, (1 + 2*ν*)*ε* represents the resistance variation arising from the change in geometrical dimensions.

#### 3.1.2. Crack Propagation and Contact-Resistance Modulation Mechanism

Crack propagation and contact-resistance modulation constitute another important mechanism by which LIG achieves highly sensitive strain responses. Under externally applied strain, the pre-existing microcracks on the surface or within the interior of LIG may further open, propagate, or close, thereby altering the local contact state between adjacent layers [67]. During stretching, crack propagation weakens the electronic coupling between neighboring sheets and increases the tunneling distance and contact resistance, resulting in a pronounced increase in device resistance. During stress release, some cracks gradually reclose, the contact resistance decreases, and the resistance signal recovers correspondingly.

### 3.2. Key Performance Metrics of LIG-Based Resistive Strain Sensors

The performance of LIG-based flexible strain sensors is generally evaluated in terms of sensitivity, detection range, linearity, response and recovery time, as well as stability and durability.

Among these parameters, sensitivity is the core indicator used to characterize the response capability of a device to strain stimuli. It is usually quantified by the ratio of normalized resistance change to applied strain, namely the GF, which is commonly expressed as follows [68,69]:

(3) $$GF=\frac{\frac{\Delta R}{R_{0}}}{\varepsilon }=\frac{R-R_{0}}{R_{0}\varepsilon }$$

where *R*_0_ is the initial resistance of the sensor, *R* is the resistance under the applied strain, Δ*R* is the change in resistance, and ε is the applied strain. A higher GF indicates that the device is more sensitive to deformation.

The detection range describes the strain interval over which the sensor can maintain a stable response. In general, the ability to detect small strains is closely related to interfacial microstructural variations and the sensitivity of local cracks. The capability to operate under large strains, by contrast, depends more on the deformability of the material structure, the flexibility of the substrate, and the ability of the conductive network to remain intact under substantial deformation. Linearity reflects the proportional relationship between the output signal and the applied strain and is therefore an important indicator for evaluating the quantitative sensing capability of the device. The response and recovery times represent the dynamic tracking capability of the sensor toward external stimuli, whereas stability and durability determine whether the device can operate reliably over the long term. Overall, the development of high-performance LIG strain sensors does not simply pursue the improvement of a single metric but rather requires a comprehensive balance among sensitivity, detection range, linearity, dynamic response, and cyclic stability.

### 3.3. Performance Optimization Strategies of LIG-Based Strain Sensors

#### 3.3.1. Material Compositing and Doping Modification

Material compositing and doping modification are important strategies for improving the overall performance of LIG strain sensors. Because pure LIG often cannot achieve simultaneous optimization of sensitivity, linearity, detection range, and environmental stability, researchers typically introduce functional components—such as conductive polymers, carbon-based nanomaterials, metal oxides, or elastic polymers—to regulate its conductive network structure and interfacial characteristics. On the one hand, composite modification helps optimize the internal conductive pathways of LIG and enhance the electrical response induced by strain. On the other hand, by introducing flexible phases or functional interfaces, it can also improve the deformability and environmental adaptability of the device.

#### 3.3.2. Pattern Design and Structural Optimization

**Figure 5:**
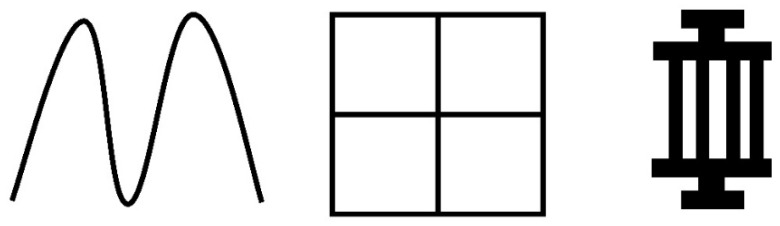
Schematic illustration of different laser patterns.

Pattern design is a key engineering strategy for improving the performance and application adaptability of LIG strain sensors. Owing to the excellent programmability and pattern-definition capability of laser processing, LIG can be readily constructed into various geometries, such as serpentine [70], grid-like, and interdigital structures [71] (Figure 5). Different patterns not only determine the length and distribution of conductive pathways but also influence the transmission, concentration, and release of external stress within the sensing layer. These factors significantly affect sensitivity, linearity, and detection range of the device.

### 3.4. Current Research Status of LIG Strain Sensors

#### 3.4.1. Film-Based and Composite-Modified LIG Strain Sensors

In recent years, research on LIG strain sensors has continued to advance, particularly in the areas of composite modification and pattern design. Gao et al. [72] proposed a multicomponent co-doped LIG strain sensor that can be directly fabricated on a stretchable elastomer. Laser processing promoted the formation of crack-like sensitive structures, thereby improving device sensitivity. The incorporation of Cu and SiO_2_ nanoparticles further enhanced the electrical response and interfacial bonding strength. As a result, the tensile strain sensor achieved a relatively wide detection range (48%) and high sensitivity, with a maximum GF of 318.5. Wang et al. [57] drew inspiration from fingerprint structures and fabricated fingerprint-patterned LIG on a PI film in one step. The film was then transferred onto an Ecoflex substrate to assemble a resistive strain sensor. The device exhibited a fast response time (~70 ms), good sensitivity, and a relatively wide strain-detection range (0–50%), reaching a GF of 191.55 in the 42–50% strain range while maintaining stable electrical responses after 1500 cycles. Lee et al. [73] proposed a ZnO nanoparticle (NP)-assisted photothermal enhancement strategy for preparing LIG, which was then transferred onto a polydimethylsiloxane substrate. Their results showed that ZnO NPs could selectively reduce the threshold fluence required for the conversion of PI into LIG. At 10% strain, the device achieved a GF of 1214, which was about 60 times higher than that of the device without ZnO nanoparticles, demonstrating a remarkable enhancement in strain-response capability. Zou et al. [74] fabricated a serpentine strain sensor based on LIG. The structure improved device stretchability and deformation adaptability, while the geometric design with local stress concentration amplified the strain-induced electrical response. These studies demonstrate that the performance trade-off between sensitivity and working range in pure LIG can be alleviated, to some extent, through composite-system design, interfacial regulation, and pattern optimization.

#### 3.4.2. Textile-Based LIG Strain Sensors

Textile-based LIG strain sensors have emerged in recent years as one of the most distinctive development directions. Compared with film-based devices, textile substrates possess inherent porous fibrous networks, good flexibility, and breathability, making them more suitable for wearable electronics and smart textile applications. Kim et al. [75] directly converted Kevlar fabric into LIG using ultrafast laser processing, thereby constructing a fast and flexible textile strain sensor. Their work demonstrated that textile substrates can serve not only as flexible support but also as effective platforms for integrating LIG conductive networks to detect dynamic signals such as heartbeat and joint motion. Yang et al. [76] further realized one-step, mask-free LIG patterning on nonwoven, knitted, and woven fabrics using a femtosecond laser. The resulting electronic textiles exhibited both high conductivity and good chemical reliability, highlighting the potential of textile-based LIG for multimodal wearable devices. Overall, textile-based LIG strain sensors show clear advantages in wearable adaptability, but their further development still depends on improvements in carbonization uniformity, interfacial reliability, and long-term stability.

To more intuitively compare the differences among various LIG flexible strain sensors in terms of material systems, device structures, and sensing performance, Table 2 summarizes representative recent studies in this field.

## 4. Research Progress in LIG-Based Resistive Temperature Sensors

### 4.1. Sensing Mechanisms of Temperature Sensors

#### 4.1.1. Positive Temperature Coefficient

**Figure 6:**
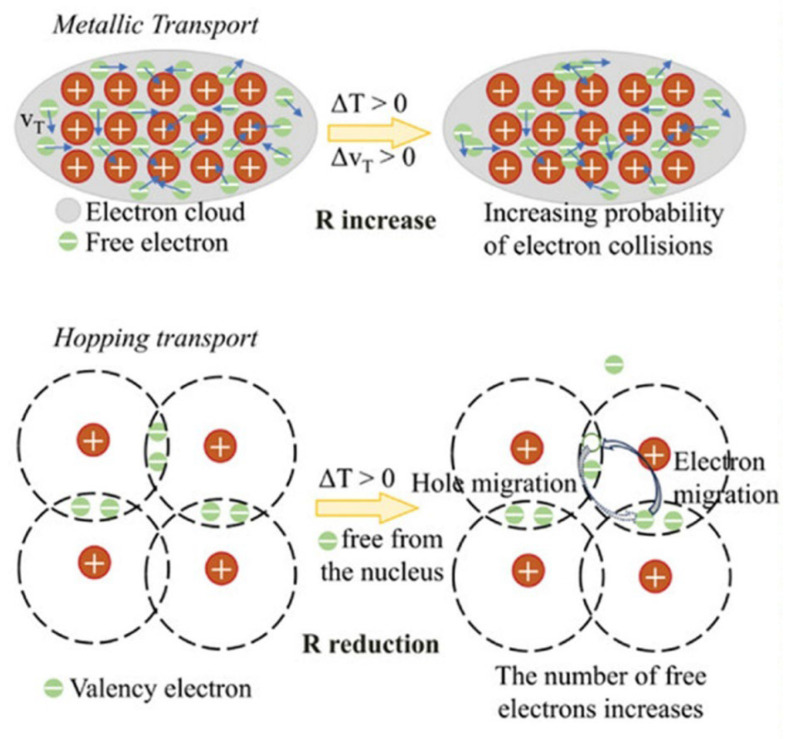
Schematic illustration of the working mechanism of the resistive temperature sensor [80].

The response of resistive temperature sensors can generally be classified into two types: positive temperature coefficient (PTC) and negative temperature coefficient (NTC) behaviors. PTC responses are commonly observed in metallic materials and certain composite conductive systems and are essentially associated with enhanced carrier scattering and weakened conductive pathways caused by increasing temperature. As shown in Figure 6, in a metallic conduction mechanism, a rise in temperature intensifies lattice thermal vibration. This increases the probability of electron collisions and hinders carrier transport, consequently increasing the electrical resistance of the material. For composite systems containing polymer matrices, temperature elevation may also induce thermal expansion of the matrix. This weakens the effective contact within the conductive network and leads to a more pronounced PTC characteristic. The relative change in resistance is generally expressed as follows [78]:

(4) $$\frac{\Delta R}{R_{0}}=\alpha \Delta T$$

where *α* is the temperature coefficient of resistance (TCR), which is used to characterize the sensitivity of a material’s resistance to temperature variation, and Δ*T* is the temperature change. In general, a larger TCR indicates a higher sensitivity of the material to temperature variation.

#### 4.1.2. Negative Temperature Coefficient

NTC behavior is widely observed in semiconductors, metal oxides, and carbon-based materials with abundant defects. The underlying mechanism is that increasing temperature promotes the thermal excitation and transport of charge carriers. More carriers can then participate in conduction, or they can more easily overcome localized potential barriers and defect energy levels, leading to a decrease in material resistance. As shown in Figure 6, under a hopping-transport mechanism, elevated temperature increases the number of free charge carriers and facilitates the migration of electrons or holes. As a result, the overall resistance of the material decreases. This process can usually be described by a thermally activated model as follows [79]:

(5) $$R=R_{0}e^{\frac{E_{a}}{kT}}$$

where *E*_a_ is the activation energy for thermally activated transport, *k* is the Boltzmann constant, and *T* is the absolute temperature.

### 4.2. Key Performance Metrics of LIG-Based Resistive Temperature Sensors

The performance of LIG-based temperature sensors is generally evaluated in terms of sensitivity, response and recovery characteristics, linearity, operating range, stability, and repeatability. Among these parameters, sensitivity is the core indicator used to characterize the response capability of a device to temperature variation. For resistive temperature sensors, it is usually quantified by the temperature coefficient of resistance (TCR). In general, a larger absolute value of TCR indicates a higher sensitivity of the device to temperature change.

The response and recovery times reflect the dynamic tracking capability of the device toward thermal stimuli and are usually closely related to the thickness of the sensing layer, heat-transfer efficiency, and interfacial contact state. Linearity is used to describe the correspondence between the output signal and temperature variation; high linearity is beneficial for improving measurement accuracy and simplifying subsequent signal processing. The operating range determines the applicable temperature interval of the device and is therefore an important indicator for evaluating its suitability for specific application scenarios.

In addition, stability and repeatability are also important parameters for evaluating the performance of temperature sensors. The former is mainly reflected in signal drift and environmental adaptability during long-term operation, whereas the latter describes the consistency of the device response over repeated heating/cooling cycles. For LIG-based flexible temperature sensors, the temperature response is typically governed by multiple factors, including defect states, localized energy barriers, porous conductive networks, and heterointerfaces. As a result, different performance metrics often exhibit synergistic or competitive relationships; for example, an increase in sensitivity may be accompanied by a decrease in linearity or stability. Therefore, the design of high-performance LIG temperature sensors should not only focus on improving a single parameter, but should also emphasize a comprehensive balance among sensitivity, response speed, linearity, and long-term reliability. Such a balanced design is essential to meet the requirements of applications such as health monitoring, electronic skin, and wearable devices, where high accuracy, rapid response, and high stability are simultaneously required.

### 4.3. Performance Optimization Strategies of LIG-Based Resistive Temperature Sensors

To improve the overall performance of LIG-based resistive temperature sensors, the functional components, such as metal oxides, metal nanoparticles, carbon-based nanomaterials, or conductive polymers, can effectively regulate defect density, carrier concentration, and interfacial barriers of LIG. This regulation, in turn, improves the temperature-response behavior of the device. On the one hand, the incorporation of semiconducting or metal-oxide components helps enhance thermally activated transport effects and enlarge the magnitude of resistance variation with temperature. On the other hand, carbon-based materials or metallic components can further optimize conductive pathways, reduce interfacial transport resistance, and improve the signal stability and repeatability of the device.

### 4.4. Current Research Status of Laser-Induced Graphene Temperature Sensors

In recent years, continuous efforts have been devoted to improving the sensitivity, linearity, and stability of LIG temperature sensors, among which composite design has become one of the most adopted strategies. By introducing metals, metal oxides, carbon materials, or other functional components into pure LIG systems, researchers can effectively regulate the carrier-transport behavior, interfacial barriers, and localized conductive pathways, thereby improving temperature-sensing performance. At present, the composite design of LIG temperature sensors is mainly focused on three categories: metal/metal oxide composites, carbon-based composites, and composites with other functional materials.

In metal/metal oxide composite systems, Chen et al. [81] reported a laser-induced nickel-based porous graphene (NiO/LIG) temperature sensor, which exhibited a high sensitivity of −1.91%·°C^−1^ together with rapid response and recovery behavior. Bai et al. [82] prepared a Cu-LIG composite with a TCR of 1.1029%·°C^−1^, which showed rapid response within the range of 30–90 °C and excellent cyclic stability (>17,000 s). These results indicate that the introduction of metals or metal oxides not only provides additional thermosensitive response units but also optimizes carrier-transport pathways through the heterointerfaces formed with LIG, effectively enhancing temperature sensitivity.

Carbon-based composites also represent an important direction in the development of LIG temperature sensors. Carbon materials such as reduced graphene oxide have been widely used in flexible temperature sensors, mainly because they can improve the continuity of conductive networks, regulate the distribution of defects and localized states, and enhance flexibility, adaptability and cyclic stability. Han et al. [83] constructed a temperature sensor composed of a reduced graphene oxide (rGO) temperature-sensitive layer and flexible LIG interdigital electrodes. Benefiting from the negative temperature coefficient behavior of both rGO and LIG, the sensor exhibited a high sensitivity of −1.56%·°C^−1^ in the range of 25–45 °C, with a resolution of 0.2 °C. Compared with metal-oxide composite systems, carbon-based composites place greater emphasis on the synergistic optimization of conductive-network continuity and structural flexibility and therefore show certain advantages in maintaining device flexibility, improving cyclic stability, and enhancing interfacial compatibility.

In addition to the above systems, composites with other functional materials also provide new design strategies for optimizing the performance of LIG temperature sensors. Li et al. [84] investigated the temperature-response behavior of LIG transferred onto a stretchable elastomer and found that its TCR was approximately −0.44%·°C^−1^. Hui et al. [85] developed a siloxene/LIG composite temperature sensor, which exhibited a stable linear resistance response with a sensitivity of up to 0.139%·°C^−1^. Overall, composite strategies can effectively overcome the limitations of pure LIG systems in terms of sensitivity and linearity by regulating heterointerfaces, reconstructing localized energy barriers, and enabling multiphase synergistic conduction.

To systematically compare the material design, operating temperature range, and key performance parameters of different LIG-based flexible temperature sensors, Table 3 summarizes representative studies reported in recent years.

## 5. Challenges and Future Perspectives for LIG Sensors

### 5.1. Current Challenges and Bottlenecks

Although LIG has demonstrated significant advantages in the field of flexible sensors, its practical application still faces several key challenges. First, the material uniformity and fabrication reproducibility of LIG still need to be further improved. Slight fluctuations in local energy distribution during laser processing, variations in precursor microstructure, and changes in environmental conditions such as humidity and oxygen concentration often lead to differences in graphitization degree, defect density, and pore structure. These differences in turn result in nonuniform electrical properties [87]. In addition, LIG fabrication is highly sensitive to parameters such as laser power, scanning speed, focal position, and precursor state. Differences in molecular weight, additive composition, and thermal stability among polymer films from different sources may further cause variations in LIG morphology and conductivity, even under the same processing conditions [88]. Such structural and performance dispersion affects the stability of sensor signal output and limits the calibration and integration of multichannel devices and array-based systems. It also increases the difficulty of stable batch-to-batch manufacturing. Although some studies have attempted to improve consistency and reproducibility by establishing standardized process windows or introducing in situ feedback regulation, there is still a lack of universal manufacturing protocols applicable for diverse substrates and application scenarios [89].

Second, as LIG evolves from single-function sensing toward multifunctional integration, multisignal coupling and cross-interference have become increasingly prominent. When LIG is simultaneously used for detecting signals such as strain and temperature, different external stimuli may produce similar resistance responses, thereby increasing the difficulty of signal decoupling. For example, in wearable health monitoring, mechanical deformation caused by body motion is often accompanied by local temperature variation. When these two factors act on the sensor simultaneously, the output signals can easily overlap and compromise the accuracy of parameter identification. Although efforts have been made to mitigate such coupling interference through functional partition design, temperature-compensation structures, introduction of thermoelectric responses, and signal-processing methods, multiparameter cooperative sensing systems that combine both high accuracy and broad applicability still require further development [90,91,92]. Therefore, achieving efficient signal separation, accurate identification, and stable output under multiple physical-field stimuli remains one of the core issues for the further development of multifunctional LIG sensors.

In addition, the large-scale system integration of LIG still faces challenges related to process compatibility and packaging reliability. Although roll-to-roll laser processing and high-speed scanning technologies enable large-area fabrication, efficiently integrating LIG with flexible circuits, wireless communication modules, and energy units, while achieving stable electrical interconnection and environmental protection, remains a critical bottleneck for practical application. In particular, under harsh conditions such as high humidity, elevated temperature, and repeated bending, issues including interfacial failure between LIG and the encapsulation layer, electrode damage, and performance degradation are still rather prominent.

Overall, the further development of LIG-based flexible sensors still requires coordinated breakthroughs in material uniformity control, manufacturing standardization, multisignal decoupling, and integration and packaging reliability, so as to lay the foundation for their practical and industrial applications.

### 5.2. Future Perspectives

Looking ahead, the development of LIG in the field of flexible sensing will mainly revolve around three directions: higher integration, greater intelligence, and enhanced sustainability. It is also expected to demonstrate broader application potential in electronic skin, health monitoring, soft robotics, and intelligent sensing systems.

First, LIG is expected to advance toward highly integrated, multimodal, and high-density sensing arrays. Owing to its excellent flexibility, convenient patterning capability, and good compatibility with flexible substrates, LIG is highly promising as a key material for constructing high-density and multimodal sensing arrays. Through microstructural design and functional modification, LIG can achieve coordinated responses to multiple stimuli, such as pressure, strain, and temperature, thereby providing a material basis for biomimetic sensing and human–machine interaction. Zhang et al. [93] prepared porous thermoelectric materials based on LIG, enabling decoupled detection of both temperature and strain signals. Future studies should further optimize micro/nanostructures and interfacial engineering to improve the spatial resolution and multimodal integration capability of such arrays, promoting the development of LIG toward large-area and high-density integration.

To address the key bottlenecks of material uniformity and signal decoupling, both experimental and computational strategies can be pursued. Uniformity can be improved through precise control of laser parameters (e.g., power, scanning speed, and focal distance) [94]. Complementary approaches include the optimization of precursor formulations, in situ monitoring, and the use of high-precision lasers such as femtosecond or ultraviolet sources [95]. Machine-learning-assisted parameter tuning has also shown promise for rapidly identifying suitable processing windows [96]. Chen et al. [97] applied LIG-embedded sensors in composite structures combined with machine learning to monitor impact damage. For signal decoupling, effective strategies include microstructural partitioning, independent multichannel readout, and circuit isolation. In parallel, computational approaches, such as finite element analysis and multiphysics modeling, can predict and optimize microstructure layouts, stress distribution, and conductive network evolution, guiding experimental design. Han et al. [98] reported layered structures combining a laser-induced graphene/silicone rubber (LIG/SR) pressure layer with a NiO temperature layer, achieving simultaneous pressure and thermal detection with minimal crosstalk. Collectively, these strategies are expected to progressively resolve the above challenges, enabling scalable deployment of LIG-based sensors.

Second, LIG is expected to evolve from simple signal acquisition toward intelligent diagnosis and active feedback. In wearable health-monitoring applications, LIG devices can not only monitor physiological signals such as heart rate and respiration in real time but also integrate with flexible energy units and wireless communication modules to construct self-powered and intelligent monitoring platforms. With the continuous advancement of mobile healthcare and smart terminal technologies, flexible wearable sensors are expected to be integrated with smartphones and other handheld devices. Such integration facilitates real-time acquisition and intelligent classification of skin lesion images, thereby exhibiting great application potential in health monitoring and personalized medicine [99]. When further combined with artificial-intelligence-driven data analysis methods, LIG devices may progress from mere signal collection to data diagnosis and active feedback. In soft robotics and intelligent sensing systems, LIG also enables conductive patterns to be constructed in situ on elastic substrates, avoiding complicated transfer processes and maintaining structural integrity and electrical stability under large deformation. Through regional functionalization, multilayer structural design, and optimization of complex patterns, LIG will endow devices with more advanced capabilities for contact recognition and environmental perception, facilitating skin-like intelligent interaction.

LIG is also expected to promote green manufacturing and the development of degradable electronic devices. Its fabrication process generally does not require complex wet-chemical reagents, masks, or high-vacuum conditions and can also be combined with biomass precursors such as paper and wood to realize low-cost and relatively environmentally friendly processing routes. Future efforts should focus on developing degradable, recyclable, and sustainably manufactured LIG-based flexible devices, driving their evolution toward low-carbon and environmentally responsible technologies to meet the demands of next-generation green electronics.

Overall, with the continued advancement of material design, device construction, and data-processing technologies, LIG-based flexible sensors are expected to further evolve toward higher performance, multifunctionality, and system-level integration. Coordinated breakthroughs in controllable material fabrication, precise structural design, cross-scale integrated manufacturing, and intelligent signal processing are essential to advance LIG-based flexible sensors from laboratory research to large-scale practical applications.

## Figures and Tables

**Table 2:** Representative recent studies and performance comparison of LIG-based flexible strain sensors.

Sensing Material/Device Structure	GF	Detection Range	Response/Recovery Time/ms	Number of Cycles	Reference
Cu- and SiO_2_-co-doped crack-based LIG	318.5	0–48%	230/300	8000	[72]
LIG/Ecoflex	191.55	0–50%	70/-	1500	[57]
ZnO NPs/LIG/PDMS	1214	0–10%	200/200	2000	[73]
Serpentine-structured LIG	107.8	0–8%	-	1000	[74]
Kevlar fabric-based LIG	185.2	0–7%	180/290	1200	[75]
Textile-based LIG	117.9	0–3%	192/177	1000	[76]
LIG/PDMS	15.79	0–20%	1160/1163	2000	[77]

**Table 3:** Representative recent studies and performance comparison of LIG-based flexible temperature sensors.

Sensing Material	Operating Temperature Range/°C	TCR/%·°C^−1^	Reference
NiO/LIG	25–90	−1.91	[81]
Cu/LIG	30–90	1.1029	[82]
rGO/LIG	25–45	−1.56	[83]
LIG/PDMS	30–80	−0.44	[84]
Siloxene/LIG	25–55	0.139	[85]
LIG	−30–10	−0.044	[86]

## Data Availability

No new data were created or analyzed in this study. Data sharing is not applicable to this article.

## Abbreviations

The following abbreviations are used in this manuscript:

LIG	Laser-induced graphene
PI	Polyimide
PDMS	Polydimethylsiloxane
PEEK	Polyether ether ketone
PAN	Polyacrylonitrile
PET	Polyethylene terephthalate
TPU	Thermoplastic polyurethane
TPC	Positive temperature coefficient
NTC	Negative temperature coefficient
TCR	Temperature coefficient of resistance
